# SURVIVAL AND PERIOPERATIVE MORBIDITY OF TOTALLY LAPAROSCOPIC VERSUS OPEN GASTRECTOMY FOR EARLY GASTRIC CANCER: ANALYSIS FROM A SINGLE LATIN AMERICAN CENTRE

**DOI:** 10.1590/0102-672020180001e1413

**Published:** 2019-01-07

**Authors:** Enrique NORERO, Catalina VARGAS, Pablo ACHURRA, Marco CERONI, Ricardo MEJIA, Cristian MARTINEZ, Rodrigo MUÑOZ, Paulina GONZALEZ, Alfonso CALVO, Alfonso DÍAZ

**Affiliations:** 1 Esophagogastric Surgery Unit, Digestive Surgery Department, Hospital Dr. Sotero del Rio, Pontificia Universidad Católica de Chile, Santiago, Chile.

**Keywords:** Stomach neoplasms, Laparoscopy, Morbidity, Gastrectomy., Neoplasias gástricas, Laparoscopia, Epidemiologia, Gastrectomia

## Abstract

**Background:**

Laparoscopic gastrectomy has numerous perioperative advantages, but the long-term survival of patients after this procedure has been less studied.

**Aim:**

To compare survival, oncologic and perioperative outcomes between completely laparoscopic vs. open gastrectomy for early gastric cancer.

**Methods:**

This study was retrospective, and our main outcomes were the overall and disease-specific 5-year survival, lymph node count and R0 resection rate. Our secondary outcome was postoperative morbidity.

**Results:**

Were included 116 patients (59% men, age 68 years, comorbidities 73%, BMI 25) who underwent 50 laparoscopic gastrectomies and 66 open gastrectomies. The demographic characteristics, tumour location, type of surgery, extent of lymph node dissection and stage did not significantly differ between groups. The overall complication rate was similar in both groups (40% vs. 28%, p=ns), and complications graded at least Clavien 2 (36% vs. 18%, p=0.03), respiratory (9% vs. 0%, p=0.03) and wound-abdominal wall complications (12% vs. 0%, p=0.009) were significantly lower after laparoscopic gastrectomy. The lymph node count (21 vs. 23 nodes; p=ns) and R0 resection rate (100% vs. 96%; p=ns) did not significantly differ between groups. The 5-year overall survival (84% vs. 87%, p=0.31) and disease-specific survival (93% vs. 98%, p=0.20) did not significantly differ between the laparoscopic and open gastrectomy groups.

**Conclusion:**

The results of this study support similar oncologic outcome and long-term survival for patients with early gastric cancer after laparoscopic gastrectomy and open gastrectomy. In addition, the laparoscopic approach is associated with less severe morbidity and a lower occurrence of respiratory and wound-abdominal wall complications.

## RESUMO

**Racional:**

A gastrectomia laparoscópica tem numerosas vantagens perioperatórias, mas a sobrevivência em longo prazo após este procedimento tem sido menos estudada.

**Objetivo:**

Comparar resultados de sobrevivência, oncológica e perioperatória entre a gastrectomia completamente laparoscópica vs. aberta para câncer gástrico precoce.

**Método:**

Este estudo foi retrospectivo e os principais resultados foram a sobrevivência global e específica de cinco anos, contagem de linfonodos e taxa de ressecção R0. Resultado secundário foi a morbidade pós-operatória.

**Resultados:**

Foram incluídos 116 pacientes (59% homens, idade 68 anos, comorbidades 73%, IMC 25) que foram submetidos a 50 gastrectomias laparoscópicas e 66 gastrectomias abertas. As características demográficas, a localização do tumor, o tipo de operação, a extensão da dissecção dos linfonodos e do estágio não diferiram significativamente entre os grupos. A taxa geral de complicações foi semelhante em ambos os grupos (40% vs. 28%, p=ns) e complicações classificadas Clavien 2 (36% vs. 18%, p=0,03), respiratórias (9% vs. 0%, p=0,03) e as da parede abdominal (12% vs. 0%, p=0,009) foram significativamente menores após a gastrectomia laparoscópica. A contagem de linfonodos (21 contra 23, p=ns) e a taxa de ressecção R0 (100% vs. 96%; p=ns) não diferiram significativamente entre os grupos. A sobrevida global de cinco anos (84% vs. 87%, p=0,31) e a sobrevida específica (93% vs. 98%, p=0,20) não diferiram significativamente entre os grupos de gastrectomia laparoscópica e aberta.

**Conclusão:**

Estes resultados suportam resultados oncológicos similares e sobrevida em longo prazo para pacientes com câncer gástrico precoce após gastrectomia laparoscópica e gastrectomia aberta. Além disso, a abordagem laparoscópica está associada com morbidade menos grave e menor ocorrência de complicações respiratórias e da parede abdominal.

## INTRODUCTION

Since the first laparoscopic gastrectomy (LG) was performed in 1994 by Kitano^17^, the application of minimally invasive surgery to treat gastric cancer has exponentially increased^3^ ^,^ ^29^. Most publications originate from Asian countries^15^, and reports seldom originate from South America^23^ ^,^ ^30^.

Due to an early diagnosis program associated with our hospital^4^, we have operated on a significant number of patients with EGC over the past decade, and we began performing LG for the treatment of EGC ten years ago^24^. The current paper represents our mature single-centre experience in performing gastrectomy for EGC.

The aim of this study was to compare survival and perioperative outcomes between completely laparoscopic and open gastrectomy (OG) for EGC. Our main outcomes were overall and disease-specific survival, lymph node count and R0 resection rate. Our secondary outcome was postoperative morbidity.

## METHODS

The local ethics committee approved the study, and the informed consent of patients was waived because of the retrospective nature of the study.

This study describes a retrospective comparative study that included all consecutive patients treated with gastrectomy for EGC on final pathology at a single centre from 2006 to 2016. Patients who met the inclusion criteria for endoscopic resection were treated by endoscopic submucosal dissection and were excluded from this study^6^. A dedicated nurse prospectively collected the data. The decision between LG and OG was based on surgeon preference and experience. All surgeries were performed by attending surgeons who were experienced in open gastrectomy with a developing learning curve for LG.

The preoperative evaluation included an upper gastrointestinal endoscopy, biopsy, complete blood count, liver-function tests, electrocardiogram, and nutritional evaluation. Preoperative imaging included a thorax-abdomen-pelvis CT-scan. 

Total or distal subtotal gastrectomy was performed depending on tumour location. Lymph node dissection was performed in both groups according to the Japanese guidelines^2^.

### Laparoscopic surgical technique

Our LG technique has been previously described^24^. Briefly, a pneumoperitoneum with CO_2_ at 15 mmHg was established, and six laparoscopic ports and a 30º scope were utilized. The duodenum is divided using a 60 mm linear stapler. The oesophagus or the stomach was also divided using a 60 mm linear stapler, and the surgical specimen was extracted through a 4 cm suprapubic incision. An intracorporeal mechanical gastro-jejunostomy was performed after distal subtotal gastrectomy, and an oesophago-jejunostomy (EJ) was performed with a Roux-en-Y reconstruction using three EJ methods (Hand-sewn 23 cases^25^, orvil 2 cases and lineal stapler 1 case).

### Open surgical technique

Our OG technique has been previously described^26^. Briefly, epidural analgesia was routinely used, and a mid-line laparotomy was utilized. The same vessel-sealing device used in LG was used in open surgery. The stomach was divided using a 60 mm linear stapler, and the oesophagus was sectioned and prepared for EJ anastomosis. A hand-sewn gastro-jejunostomy or mechanical circular stapler EJ was performed.

In the postoperative period, immediate extubation was favoured, and patients began physical and respiratory therapy as soon as possible. Epidural analgesia was generally maintained for three days in OG, and a nasogastric tube was kept in place for 3-5 days after subtotal gastrectomy. An oral contrast study was performed 3-7 days after a total gastrectomy. The patients were discharged when they were able to tolerate a soft diet for 24 h. 

All deviations from a normal postoperative course of elective gastrectomy for up to 30 days or during the hospital stay were considered postoperative complications. Readmission was considered for up to 60 postoperative days. The appearance of contrast outside the EJ anastomosis in an oral contrast study or CT-scan or by direct evaluation at reoperation was defined as an EJ leak. The impossibility to advance a standard gastroscope through the anastomosis or the need for endoscopic dilation was defined as EJ stenosis. The need to maintain the nasogastric tube for over 10 days after a subtotal gastrectomy with an output exceeding 200 ml was defined as delayed gastric emptying. Abdominal wall and wound complications were both added as a composite outcome. Respiratory symptoms accompanied by an imaging study with pulmonary infiltrates were defined as pneumonia, whereas pleural effusion was characterized by these observations on imaging studies and required either a pleurocentesis or a pleurostomy. Pneumonia, pleural effusion and respiratory failure were added as composite outcomes. Complications were evaluated according to the Clavien-Dindo classification^5^.

Staging was based on the 7^th^ edition of TNM-AJCC^22^. The follow-up program consisted of a physical examination, laboratory blood tests, endoscopy, and ultrasonography or computed tomography.

### Statistical analyses

Statistical analyses were performed using SPSS version 22, Inc., Chicago, IL and Minitab 15. Categorical variables are expressed in percentages (%); quantitative values are expressed as the median (range). The Chi-squared test was used to compare frequencies, and the Mann-Whitney U test was used to compare quantitative values. Survival curves were estimated according to the Kaplan-Meier method. The log-rank test was used to compare survival curves. Patients in the LG group who were converted to open surgery remained in the LG group on an intention-treat-basis. All statistical tests were two-sided, and a p value <0.05 was considered significant.

## RESULTS

The study included 116 patients with EGC whose median age was 68 (33-86) years and median BMI was 25 (19-38). Fifty-nine percent of the cohort was male, and 73% of patients had at least one comorbidity. Moreover, 54% of patients had a previous laparotomy. Fifty patients (43%) received a LG, and 66 (57%) patients underwent an OG. The age, sex, BMI, comorbidities, surgical risk score and history of a previous laparotomy and demographic characteristics did not significantly differ between groups (Table 1).

**TABLE 1:** Patient demographics and surgery details

	Open gastrectomy l(n=66)	Laparoscopic gastrectomy (n=50)	p
Age (years)	69 (33-86)	69 (38-85)	0.618
Sex (male)	43 (65.2%)	26 (52.0%)	0.153
BMI (kg/mt2)	23.9 (19-38)	26.5 (20-32)	0.126
ASA score			0.173
I	14 (21.2%)	17 (34.0%)	
II	39 (59.1%)	28 (56.0%)	
III	13 (19.7%)	5 (10.0%)	
Hypertension	30 (45.5%)	25 (50.0%)	0.627
Diabetes	12 (18.2%)	6 (12.0%)	0.362
Cardiac disease	9 (13.6%)	5 (10.0%)	0.552
Stroke	4 (6.1%)	2 (4.0%)	0.479
Pulmonary disease	6 (9.1%)	4 (8%)	0.555
Chronic liver disease	2 (3.0%)	1 (2.0%)	0.604
Previous laparotomy	31 (50.8%)	29 (58.0%)	0.450
Upper abdominal laparotomy	20 (32.8%)	16 (32.0%)	0.930
Tumour location			0.572
Superior	23 (38.3%)	17 (34.0%)	
Middle	21 (35.0%)	15 (30.0%)	
Inferior	16 (26.7%)	18 (36.0%)	
Gastrectomy type			0.785
Total	36 (54.5%)	26 (52.0%)	
Subtotal distal	30 (45.5%)	24 (48.0%)	
Lymph node dissection			0.170
D0	0 (0%)	1 (2.0%)	
D1	8 (14.5%)	3 (6.0%)	
D1 +	12 (21.8%)	18 (36.0%)	
D2	35 (63.6%)	28 (56.0%)	
Subtotal gastrectomy reconstruction			0.005
Billroth I	0 (0%)	1 (4.8%)	
Billroth II	16 (64.0%)	4 (19.0%)	
Y Roux	9 (36.0%)	17 (76.2%)	
Operative time (min)	240 (120-480)	330 (210-510)	0.0001
Operative bleeding (cc)	300 (50-800)	110 (10-500)	0.038

BMI=body mass index, ASA=American Society of Anaesthesiologists Physical Status Classification

The location of the tumour within the stomach was similar in both groups: in 36% of patients, the tumour was located in the upper third of the stomach, whereas it was located in the middle third and distal third of the stomach in 32% and 30% of patients, respectively (Table 1). In 53% of patients, a total gastrectomy was performed, and 60% of patients had a D2 lymphadenectomy. The extent of gastrectomy and lymph node dissection did not significantly differ between groups (Table 1). The reconstruction method more frequently employed after a subtotal gastrectomy was a Roux-en-Y in LG (76%) and Billroth II in OG (64%) (p=0.005, Table 1).

The median estimated intra-operative bleeding was higher in the OG group (300 ml vs. 100 ml; p=0.038), whereas the operative time was significantly longer in the LG group (250 min vs. 330 min; p=0.0001) (Table 1). Moreover, four patients (8%) were converted to open surgery due to the misfiring of the duodenal stapler, central obesity, a cholecystoduodenal fistula, and intestinal malrotation.

The overall rate of postoperative morbidity was 35%, and the complication rate tended to be lower in the LG group, as demonstrated by a rate of 28% compared to a rate of 40% in the OG group; however, this difference was not significant (p=ns). The rates of intrabdominal or medical complications did not significantly differ between groups (p=ns). Wound and abdominal wall complications were significantly lower in the LG group (12% vs. 0%; p=0.009). In the OG group, five patients (7%) exhibited abdominal wall dehiscence, and three (4%) developed surgical site infection. Two of the five patients with abdominal wall dehiscence required a re-operation with general anaesthesia to close the abdominal wall. Respiratory complications were also significantly lower in the LG group (9% vs. 0%; p=0.031, Table 2) and were stratified as follows: 6% of patients developed pneumonia, 3% pleural effusion and one patient exhibited respiratory failure in the OG group.

**TABLE 2:** Postoperative clinical outcome and complications

Complications	Open gastrectomy l(n=66)	Laparoscopic gastrectomy (n=50)	p
Intrabdominal	10 (15.2%)	11 (22.0%)	0.343
EJ fistula	2 (5.6%)	3 (11.5%)	0.346
Duodenal fistula	0 (0%)	3 (6.0%)	0.077
Subphrenic abscess	1 (1.5%)	1 (2.0%)	0.678
EJ stenosis	2 (5.6%)	0 (0%)	0.333
Pancreatic fistula	0 (0%)	1 (0%)	0.431
Abdominal bleeding	1 (1.5%)	0 (0%)	0.569
Ileus	1 (1.5%)	0 (0%)	0.569
Delayed gastric emptying	0 (0%)	3 (12.5%)	0.082
Wound - Abdominal wall	8 (12.1%)	0 (0%)	0.009
Dehiscence	5 (7.6%)	0 (0%)	0.056
Surgical site infection	3 (4.5%)	0 (0%)	0.181
Medical	11 (16.7%)	4 (8.0%)	0.136
Respiratory	6 (9.1%)	0 (0%)	0.031
Pneumonia	4 (6.1%)	0 (0%)	0.101
Pleural effusion	2 (3.0%)	0 (0%)	0.322
Respiratory failure	1 (1.5%)	0 (%)	0.569
Arrhythmia	2 (3.0%)	0 (0%)	0.322
Pseudomembranous colitis1 (1.5%)	1 (1.5%)	2 (4.0%)	0.396
Urinary tract infection	2 (3.0%)	1 (2.0%)	0.604
Central catheter infection	1 (1.5%)	0 (0%)	0.569
Thromboembolic disease	2 (3.0%)	1 (2.0%)	0.604
Stroke	1 (1.5%)	0 (0%)	0.569
Morbidity	27 (40.9%)	14 (28.0%)	0.150
Reoperation	3 (4.5%)	2 (4%)	0.630
Morbidity Clavien ≥ 2	24 (36.4%)	9 (18%)	0.030
Morbidity Clavien ≥ 3	9 (13.6%)	2 (4.0%)	0.072
Mortality	2 (3.0%)	1 (2.0%)	0.604
Length of stay (days)	9 (5-60)	7 (4-37)	0.017
Readmission	7 (10.6%)	2 (4.0%)	0.070

In the OG and LG groups, 36% and 18% of patients developed complications classified as Clavien 2 or higher, respectively (p=0.03, Table 2). Five required a reoperation; in the OG group, two reoperation because of abdominal wall dehiscence and one due to EJ leak. In the LG group, one patient required reoperation because of an EJ leak and one due to a duodenal stump fistula. The reoperation rate did not significantly differ between groups (4.4% vs. 4%, p=ns, Table 2).

The postoperative mortality rate was 2.6% and was represented by two (3%) patients in the OG group and one (2%) in the LG group (p=ns, Table 2). The mortality causes in these three patients were pneumonia associated with postoperative stroke, pulmonary embolism and EJ leak.

The length of stay was significantly shorter by two days in the LG group (9 vs. 7 days, p=0.017, Table 2).

Fifty-seven percent of cancers were mucosal cancers, and 43% exhibited submucosal involvement; these rates did not differ between groups (p=ns). Ninety-four percent of patients were classified as N0 disease, and this rate did not differ between groups (p=ns). Moreover, two (3%) and five (10%) patients with T1A and T1B disease, respectively, exhibited lymph node metastases. The median number of lymph nodes resected was similar in the OG and LG groups (21 vs. 23; p=ns), and microscopic tumour-free margins were comparable in both groups (100% vs. 96%, p=ns, Table 3). The two patients in the LG group with a positive margin had undergone a subtotal gastrectomy for a large EGC at the beginning of the experience with LG; both patients were converted to a laparoscopic total gastrectomy three months after the initial surgery, and there was no tumour in this second resection.

**TABLE 3:** Pathologic staging, lymph node count and margin status

Complications	Open gastrectomy l(n=66)	Laparoscopic gastrectomy (n=50)	p
T			0.334
T1a (mucosa)	35 (53.0%)	31 (62.0%)	
T1b (submucosa)	31 (47.0%)	19 (38.0%)	
N			0.418
0	63 (95.5%)	46 (92.0%)	
1	0 (0%)	2 (4.0%)	
2	2 (3.0%)	1 (2.0%)	
3	1 (1.5%)	1 (2.0%)	
Stage			0.418
IA	63 (95.5%)	46 (92.0%)	
IB	0 (0%)	2 (4.0%)	
IIA	2 (3.0%)	1 (2.0%)	
IIB	1 (1.5%)	1 (2.0%)	
Lymph node count	21 (1-56)	23 (4-103)	0.300
Lymph node count			0.495
0-14	15 (22.7%)	11 (22.0%)	
15-24	26 (39.4%)	24 (30.0%)	
25 or more	25 (37.9%)	24 (48.0%)	
Margin status			0.184
R0	66 (100%)	48 (96.0%)	
R1	0 (0%)	2 (4.0%)	

**FIGURE 1:**
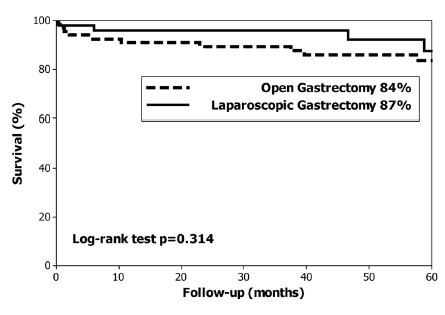
Overall 5-year survival in patients with early gastric cancer treated with open and laparoscopic gastrectomy

**FIGURE 2:**
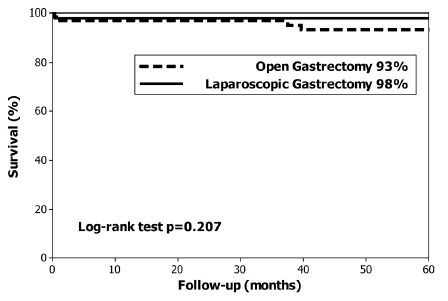
Disease-specific 5-year survival in patients with early gastric cancer treated with open and laparoscopic gastrectomy

The median follow up was 59 months. At the end of the study 96 (83%) patients were alive and 20 (17%) patients had died; specifically three (2.5%) had died of EGC recurrence, two T1A and one T1B, and none had lymph node metastases. The overall 5-year survival rate was 85% (median not reached), 84% in the OG group and 87% in the LG group (p=0.314, Figure 1). The disease-specific 5-year survival was 95% (median not reached). Disease-specific 5-year survival was 93% in the OG group and 98% in the LG group (p=0.207, Figure 2). The log-rank test did not identify differences in the long-term overall survival or in disease-specific survival between the LG and OG groups.

## DISCUSSION

The long-term overall survival after surgery for EGC reportedly exceeds 80%, and disease-specific survival exceeds 90% because most patients die from other diseases. However, most of these data were obtained in studies in which patients underwent OG^12^ ^,31^. Despite the fact that the first LG was performed more than two decades ago^1^, controversy still surrounds the use of LG for the treatment of gastric cancer because of the insufficient evidence in favour of its long-term oncologic outcomes.

Most studies comparing LG with OG have included a small number of patients and have focused only on the short-term and perioperative outcomes^29^, such us operative bleeding, operative time and postoperative morbidity ^8^ ^,^ ^15^. Moreover, only a few randomized controlled trials and non-randomized studies have evaluated long-term survival^1^ ^,^ ^9^ ^,^ ^14^ ^,^ ^16^. These studies have demonstrated similar long-term survival for patients with EGC treated with LG or OG^1^ ^,^ ^9^ ^,^ ^14^ ^,^ ^16^. Our study with a significant number of patients and with long-term follow-up supports a similar overall and disease-specific long-term survival.

Between two and 20% of patients with EGC have lymph node-positive disease^1^ ^,^ ^27^, making lymph node dissection an essential part of surgery. Most patients in our study had at least a D1+ dissection, and 6% had lymph node-positive disease. The lymph node count is usually considered an indicator of the completeness of lymph node dissection. In our study, the lymph node count was similar between the LG and OG groups, supporting the feasibility of performing a complete lymphadenectomy with the laparoscopic approach. Previously published results are conflicting, with some studies finding a lower^29^ or similar^10^ ^,^ ^13^ lymph node count. The R0 resection rate was the same between groups, 100% and 96%.

The three oncologic outcomes in this study, lymph node count, R0 resection and overall and disease-specific survival, support the oncologic equivalence of LG and OG for EGC.

Previous studies and meta-analyses indicate that the morbidity rate is lower for LG than OG^15^ ^,^ ^29^. In our study we could not demonstrate a difference in general morbidity, likely due to the sample size. However, respiratory and wound-abdominal wall complications were significantly lower in the LG group^**28**^. The reduction in wound-abdominal wall complications may specifically be associated with the totally laparoscopic approach in our study given our median BMI of 25 and the high comorbidity rate, whereas the lower rate of respiratory complications in the LG group may be associated with reduced postoperative pain and the early ambulation described for patients in the LG group^18^. Other studies have found similar lower wound complication rates^15^, better respiratory function results^18^ and lower respiratory complications^21^, supporting our findings.

Only a few studies have evaluated the severity of complications^13^ ^,^ ^16^. The studies by Kelly^13^ and Kim^16^ both reported a lower rate of mild complications but an equal frequency of more severe complications. Interestingly, our study identified a lower frequency of moderate-severe complications in the LG group, which has not been reported previously and may be associated with a lower rate of reoperations associated with abdominal wall complications and fewer respiratory complications, possibly due to the totally laparoscopic technique in the LG group.

Most publications describing LG originate in Asia^14^ ^,^ ^15^. In the West, LG has not been widely adopted and has been developed consistently by only a few expert centres^7^ ^,^ ^13^ ^,^ ^20^ ^,^ ^23^ ^,^ ^30^. In fact, the applicability of LG has been questioned by our Asian colleagues^9^, but our study supports the applicability of LG in countries outside Asia. Because of the higher BMI and comorbidities, and more common need for total gastrectomy in the west compared to Asia^9^ ^,^ ^14^, we hypothesize that the reduction in complications in Western countries may be even more pronounced in future studies.

In this study, we included a consecutive and significant number of patients with long-term follow-up. The data were obtained from a prospectively maintained database. Moreover, a contemporary control group was included. According to a score described by a recent meta-analysis evaluating nonrandomized trials of LG, our study has a high quality score^29^. Another strength of our study is the severity evaluation of complications with the Clavien score. In addition to these methodological aspects, we employed a totally laparoscopic technique, which has been described to be superior to the laparoscopic assisted method employed in most reported trials^11^.

Some of the limitations of this study are that the decision to perform LG or OG may have been influenced by variables such as surgeon and hospital experience at the time and a preoperative diagnosis of a higher tumour stage, despite the fact that the two groups were well balanced. Moreover, patients with EGC were included in this study on the basis of postoperative pathology and not clinical preoperative evaluation.

## CONCLUSION

The results of this study support a similar oncologic outcome and long-term survival for laparoscopic gastrectomy and open gastrectomy for patients with EGC. In addition, the laparoscopic approach is associated with less severe morbidity and a lower occurrence of respiratory and wound-abdominal wall complications.
